# Prevalence and risk factors associated with infections linked to carbapenemase-producing *Acinetobacter* species circulating in the city of Yaoundé, Cameroon

**DOI:** 10.1016/j.ijregi.2025.100698

**Published:** 2025-07-01

**Authors:** Cedric Fossi Tchinda, Karyom Djim-Adjim-Ngana, Therence Annie Mbogning, Brice Fredy Nemg Simo, Sosthene Boido Baira, Gaizirene Egoume Feudjieu, Sonia Gayap Matchuenkam, Gael Njini Nfor, Guy Roussel Nguemto Takuissu, Kikie Josiane Essola, Connie Constance Georgina Walyaro, Armelle Deutou Tchamgoue

**Affiliations:** 1 Pharmacology and Drugs Discovery Laboratory, Centre for Research on Medicinal Plants and Traditional Medicine, Institute of Medical Research and Medicinal Plants Studies (IMPM), Yaoundé, Cameroon; 2 Laboratory of Microbiology, Infectious Diseases and Immunology, Centre for Research on Health and Priority Pathologies, Institute of Medical Research and Medicinal Plant Studies, Yaoundé, Cameroon; 3 Department of Clinical biology, Estuary Academic and Strategic Institute (IUEs/INSAM), University of Douala, Douala, Cameroon; 4 Centre for Research in Infectious Disease, Yaoundé, Cameroon; 5 Kaele District Hospital, Ministry of Public Health, Kaele, Cameroon; 6 Department of Microbiology, Faculty of Science, University of Yaoundé I, Yaoundé, Cameroon; 7 Centre for Food, Food Security and Nutrition Research, Institute of Medical Research and Medicinal Plants Studies (IMPM), Yaoundé, Cameroon; 8 Faculty of Medicine and Pharmaceutical Sciences of the University of Douala, Douala, Cameroon; 9 Clinical Biology Laboratory, Laquintinie Hospital of Douala, Douala, Cameroon; 10 International Society for Infectious Diseases (ISID), European and Developing Countries Clinical Trials Partnership (EDCTP), Talk AB[M]R, Nairobi, Kenya

**Keywords:** Prevalence, Risk factors, Acinetobacter, Carbapenemase, Health facilities, Yaoundé

## Abstract

**Objectives:**

Carbapenems are critical antibiotics used against multidrug-resistant infections, yet resistance is increasing, especially among Gram-negative bacteria like *Acinetobacter* species. In Cameroon, while *Acinetobacter baumannii* is well studied, little is known about other species such as *Acinetobacter pittii, Acinetobacter nosocomialis*, and *Acinetobacter calcoaceticus*. This study assessed the prevalence of carbapenemase-producing *Acinetobacter* and associated risk factors in Yaoundé hospitals.

**Methods:**

A 6-month prospective cross-sectional study (April-September 2024) was conducted using 531 clinical samples from four health facilities. Strains were identified via morphologic, biochemical, and API 20 NE® tests. Antibiotic susceptibility was assessed using disc diffusion, and carbapenemases were characterized phenotypically.

**Results:**

Of the samples, 4.9% yielded *Acinetobacter*, with *A. baumannii* being most common (80.8%), followed by *A. calcoaceticus* (11.5%) and *Acinetobacter anitratus* (7.7%). *A. baumannii* showed high antibiotic resistance, especially to beta-lactams, with 85.71% producing carbapenemases (83.33% class B and 16.67% class A). Significant risk factors included hospitalization, current antibiotic therapy, recent antibiotic use, and age group 38-47 years.

**Conclusions:**

These findings highlight an urgent need for better surveillance, stricter antibiotic stewardship, and enhanced infection control strategies to limit the spread of resistant *Acinetobacter* infections in Yaoundé’s health care settings.

## Highlights

• Among *Acinetobacter baumannii* isolates, 85.71% were carbapenemase-producing strains.
• Resistance to all antibiotics tested was high except colistin (11.1% resistance by E-test).
• Recent hospitalization and antibiotic use were major risk factors.
• Differentiating colonization from infection is essential when interpreting these findings.

## Introduction

*Acinetobacter* is a non-fermenting Gram-negative coccobacillus known for its ability to colonize the human body and various environmental reservoirs [1]. Infections caused by these species are a growing global health concern due to their increasing antimicrobial resistance and impact on patient outcomes. A study conducted in the European Union and surrounding areas reported that carbapenem-resistant *Acinetobacter* species were responsible for approximately 27,000 illnesses and 2300 deaths in 2015, underscoring the severity of this issue [2]. Over the past three decades, *Acinetobacter*, particularly *Acinetobacter baumannii*, has emerged as a significant cause of health care-associated infections, leading to high morbidity and mortality rates, especially in immunocompromised patients, with case fatality rates ranging from 26.5-91% [3]. The prevalence of *Acinetobacter* infection varies based on the geographic location and the socioeconomic status of the patients [[4], [5], [6]].

Data show a high burden in intensive care units, with reported prevalences of 19.2% in Asia, 17.1% in Eastern Europe, 14.8% in Africa, and lower rates in other regions [6]. Several species of *Acinetobacter* can cause illness in humans, with *A. baumannii* being the most clinically significant, accounting for over 80% of infections worldwide. This species is predominantly found in hospitals but can also be isolated from soil, water, and medical facilities, and has a high mortality rate exceeding 50% [7]. *Acinetobacter spp* is mainly transmitted via environmental surfaces and the hands of health care staff, and can cause infections such as bacteremia, pneumonia, meningitis, urinary tract infections, and surgical site infections [8].

Other clinically significant species include *Acinetobacter pittii*, commonly found in children, and *Acinetobacter nosocomialis,* both associated with community-acquired and nosocomial infections [9]. Newly identified species, *Acinetobacter seifertii* and *Acinetobacter dijkshoorniae*, have been isolated from human clinical samples [10]. In contrast, *Acinetobacter calcoaceticus* is a non-pathogenic environmental bacterium isolated from soil that has been rarely implicated in disease transmission [11]. Currently, few antibiotics are effective against these pathogens [12,13]. The organism’s extensive resistance profile complicates treatment. Carbapenems are typically the last line of effective therapy, but resistance to these agents is rising, primarily due to mechanisms, such as carbapenemase production, overexpression of beta-lactamases, efflux pumps, and reduced membrane permeability [[12], [13], [14], [15]]. Multidrug-resistant and pan-drug-resistant strains are increasingly common, leaving few therapeutic options. Although various combination therapies have been studied—such as colistin-based regimens—efficacy remains limited, and patient outcomes are often poor [14]. The resistance of the latter to carbapenems is linked to several mechanisms, such as the overexpression of extended-spectrum beta-lactamases, efflux pumps, impermeability, and/or the expression of carbapenem-hydrolyzing beta-lactamases known as carbapenemase [15]. Despite growing global concern, critical knowledge gaps remain regarding the prevalence, molecular characteristics, and clinical impact of carbapenemase-producing *Acinetobacter* species, especially in under-resourced settings. These gaps hinder the implementation of effective empirical treatment protocols and infection control measures. In Africa, particularly Central Africa, data on carbapenemase-producing *Acinetobacter* species are scarce [16]. Cameroon, like many developing countries, faces challenges in monitoring antimicrobial resistance trends due to limited diagnostic capabilities. Although *A. baumannii* is recognized as a common cause of nosocomial infections in Cameroonian hospitals [16,17], there is little information on the prevalence, distribution, and risk factors associated with carbapenemase-producing strains.

A study by Djuikoue et al. [16] in four Cameroonian hospitals found that 40.12% of A. baumannii isolates produced carbapenemases, predominantly class B enzymes (62.69%). Resistance was widespread, and significant associations were found with male patients, younger age groups (10-39 years), and pus samples. Another study in Douala by Ebongue et al. [17] reported high rates of multidrug resistance, with imipenem and amikacin being the most effective remaining antibiotics. This study aims to address the critical knowledge gap by determining the prevalence of carbapenemase-producing *Acinetobacter* species and identifying associated risk factors in selected health facilities in Yaoundé, Cameroon. The findings are expected to inform empirical therapy choices and strengthen infection prevention strategies in hospital settings.

## Materials and methods

### *Study design, location, and period*

A cross-sectional study was carried out over a period of 6 months, from April 1 to September 30, 2024. The samples were collected in four reference health facilities located in the Center region and more precisely in the city of Yaoundé, which is one of the most populated cities in Cameroon. The health facilities consisted of the Military Hospital of Yaoundé, the Biyem-Assi District Hospital, the Efoulan District Hospital, and the Belle Rose Medical Centre. These facilities were selected due to their status as referral centers receiving a high volume of patients with complex pathologies. The strains’ re-identification and further biological analyses were carried out at the Microbiology Unit of the Pharmacology and Drug Discovery Laboratory of the Institute of Medical Research and Medicinal Plant Studies.

### *Sampling method and selection criteria*

During the study period, all strains belonging to one of the *Acinetobacter* species or suspected of also belonging to one of the *Acinetobacter* species isolated from pathologic specimens (pus, wounds, urines, bloods, effusions fluids and cervico-vaginal swabs, etc.) at each bacteriology laboratory of concerned the health facilities were systematically collected, stored, later included in the study for the upcoming re-identification and analyses. Strains not belonging to one of the *Acinetobacter* species after re-identification and those that lacked useful clinical information were excluded from the study. Some of the collected specimens, including urine, pus, and cervico-vaginal swabs, may reflect colonization rather than infection. This limitation was considered in the interpretation of prevalence and resistance data. No formal power calculation was performed; instead, a convenience sample was used to ensure basic representativeness for the analysis of risk factors.

### *Identification and samples processing*

#### Strain identification

The strains were grown on MacConkey agar and blood agar and then identified using morphologic and biochemical tests and the API 20 NE® gallery (BioMérieux, Marcy l'Etoile, France).

#### Antimicrobial susceptibility testing

The study of antibiotic susceptibility was performed using the disc diffusion method on Mueller-Hinton agar plates and interpreted as recommended by the antibiogram committee of the French Society of Microbiology in their 2014 recommendations. This choice is related to its local adoption by Cameroonian laboratories and the availability of technical support. The antibiotic discs tested were ticarcillin, ticarcillin-clavulanate, piperacillin, piperacillin-tazobactam, cefepime, ceftazidime, imipenem, amikacin, gentamicin, tobramycin, netilmicin, ciprofloxacin, sulfamethoxazole-trimethoprim, and colistin. The reading of the antibiograms was performed using the Open Source IRIDA-based Surveillance (OSIRIS) expert system. The resistance to colistin was confirmed by the determination of the minimum inhibitory concentrations (MICs) using the E-test method (Biomérieux, Marcy l’Etoile, France), according to the manufacturer’s recommendations. The interpretation of colistin MICs followed the 2024 EUCAST (European Committee on Antimicrobial Susceptibility Testing) guidelines, suitable for non-fermenting Gram-negative bacilli such as *A. baumannii*. E-test strips provided accurate MIC measurements. Strict quality control measures were implemented, including a reference strain (ATCC 19606) and negative controls. Results were validated only when readability conditions and control ranges were met, ensuring data reliability. These procedures are essential for producing credible susceptibility data and supporting accurate clinical interpretation.

### *Detection of carbapenemases*

All isolates of imipenem-resistant *Acinetobacter* species were subjected to the carbapenemase phenotypic characterization tests using boronic acid and chelating agent (EDTA), for identification of class A and class B (metallo-beta-lactamases [MBL]) carbapenemases, respectively. *Acinetobacter* species isolates were tested for Klebsiella pneumoniae carbapenemase carbapenemases production on disks containing boronic acid. A disk containing imipenem (Mast, UK) and another containing imipenem with 400 μg of boronic acid (Sigma-Aldrich, Germany) was placed on the Mueller-Hinton agar on which the test organism was seeded. The diameter of the growth-inhibitory zones around the imipenem disk with boronic acid was compared with that around the corresponding imipenem disk without boronic acid. The test was considered positive for the detection of class A carbapenemase production when the diameter of the growth-inhibitory zone around the imipenem disk with boronic acid was ≥5 mm larger than that around the disk containing only imipenem [18]. The detection of MBL production was performed using the same principle, based on inhibition by EDTA. This technique consisted of two imipenem disks; one with and another without 10 μl of 0.5 M EDTA. An increase of 10 mm in the inhibition zone diameter in the presence of EDTA was considered positive [19].

### *Statistical analysis*

The different data collected were recorded in Excel 2019 software and then analyzed using the statistical software, Statview 5.0 (SAS Institute Inc., Cary, NC, USA). The analysis included the calculation of the frequency and their intervals at a 95% confidence interval (CI, for qualitative variables) and the mean or the median (for quantitative variables). Odds ratios (OR) were used to determine the risk factors associated with *Acinetobacter* species-producing carbapenemases contamination. Chi-square test was used to compare the proportions of categorical variables, and a *P*-value <0.05 was considered statistically significant. Multivariate analyses were preceded by univariate analyses (χ²) to select significant variables (*P* <0.05) for inclusion in the logistic model.

### *Quality control*

To ensure the reliability of identification, antimicrobial susceptibility testing, and carbapenemase detection procedures, the *A. baumannii* ATCC 19606 strain was used as a quality control strain. This reference strain is recommended for validating antibiotic susceptibility assays according to Clinical and Laboratory Standards Institute/EUCAST standards.

## Results

### *Description of sociodemographic characteristics, clinical, and medical history of patients*

A total of 531 participants were included in this study. The majority of them were men, accounting for 54.4% (289 participants), with a sex ratio of 1.19. Patients aged between 18 and 27 years were the most represented age group (48.8%), followed by those aged 28-37 years, with a low participation of those under 18 years (2.80%). Our sample was predominantly composed of single patients (71%). The results showed that most individuals had a higher education level (68.5%), followed by secondary education (21.7%). In terms of profession, students made up the majority (59.9%), followed by traders (9.3%). The proportion of participants with a seasonal income type was very high, representing nearly 74.4% of all respondents (Table 1).

**Table 1:** Sociodemographic characteristics, clinical and, medical history of patients.

Variables	Frequency (N = 531)	%
**Sociodemographic characteristics**		
**Age group (years)**		
˂18	15	2.8
18-27	259	48.8
28-37	115	21.7
38-47	61	11.5
>48	81	15.3
**Sex**		
Female	242	45.6
Male	289	54.4
**Marital status**		
Married	119	22.5
Single	377	71
Divorced	1	0.2
In a partnership	30	5.6
Widowed	4	0.8
**Level of education**		
Unschooled	6	1.2
Primary	46	8.6
Secondary	115	21.7
University	364	68.5
**Profession**		
Farmer	4	0.8
Trader	49	9.3
Student (school)	31	5.8
Teacher	14	2.6
Beautician	1	0.2
Student (university)	318	59.9
Civil servant	31	5.8
Gendarme	2	0.4
Engineer	5	0.9
Mason	7	1.3
Housewife	48	9,0
Military	1	0.2
Motorbike taxi driver	1	0.2
Plumber	1	0,2
Police officer	3	0.6
Retired	7	1.3
Unemployed	8	1.5
**Type of income**		
Dependent on emergencies	10	1.9
Non-existent	55	10.4
Permanent	71	13.3
Seasonal	395	74.4
**Clinical characteristics**		
**Hospitalized patient (N = 531)**		
No	394	74.2
Yes	137	25.8
**If yes, which department? (N = 137)**		
Surgery	8	5.8
Maternity	4	2.9
Internal medicine	103	75.2
Intensive care unit	9	6.6
Emergency	13	9.5
**Currently on antibiotics (N = 531)**		
No	331	62.3
Yes	200	37.7
**If yes, which antibiotic? (N = 200)**		
Augmentin	20	10
Cefepime	4	2
Ceftriaxone	159	79.5
Ciprofloxacin	16	8
Mesporin	1	0.5
**Medical history**		
**Comorbidities (N = 531)**		
No	498	93.8
Yes	33	6.2
**If yes, which comorbidities? (N = 33)**		
Diabetes	21	63.6
Renal failure	4	12.1
HIV	2	6.1
Sickle cell disease	6	18.2
**Have you used antibiotics in the** **Last 3 months? (N = 531)**		
No	278	52.4
Yes	253	47.6
**If yes, which antibiotics did you use? (N = 253)**		
Augmentin	18	7.1
Bactrim	2	0.8
Cefepime	77	30.4
Cefixime	15	5.9
Ceftriaxone	58	22.9
Ciprofloxacin	68	26.8
Cotrimoxazole	8	3.3
Doxycycline	3	1.2
Levofloxacin	2	0.8
Metronidazole	1	0.4
Ofloxacin	1	0.4
**Have you been hospitalized in the last 6 months? (N = 531)**		
No	426	80.2
Yes	105	19.8
**If yes, were you exposed to invasive medical devices (catheters, ventilators, etc.) during your hospitalization? (N = 105)**		
Yes	105	100

In terms of clinical characteristics, we found that 25.8% (n = 137) were hospitalized, whereas 74.2% were not. Among the hospitalized patients, the internal medicine department was the most affected, accounting for 75.2% (n = 103) of cases. Regarding antibiotic use, 37.7% (n = 200) of patients were undergoing antibiotic therapy, whereas 62.3% (n = 331) were not taking antibiotics at the time of the study. Ceftriaxone was the most commonly prescribed antibiotic, used by 79.5% (n = 159) of treated patients (Table 1).

The majority of patients (93.8%) had no comorbidities; among the 6.2% who did, diabetes was most frequent (63.6%), followed by sickle cell disease, renal failure, and HIV. Nearly half (47.6%) had used antibiotics in the 3 months prior, mainly cefepime (30.4%), ceftriaxone (22.9%), and ciprofloxacin (16.9%). Other antibiotics included augmentin, cefixime, and cotrimoxazole. In addition, 19.8% had been hospitalized in the past 6 months and were exposed to invasive devices such as catheters and ventilators (Table 1). Table 1 suggests that a significant proportion of *Acinetobacter* isolates may reflect community acquisition or colonization, which should be interpreted cautiously when assessing pathogenic significance.

### *Distribution of patients by type of biological sampling*

Out of the 531 biological samples collected from patients, the majority (35.8%) were from urine cultures (ECBU), followed by urethral swabs (13.4%) and cervico-vaginal swabs (11.9%), together representing more than 50% of the total sample.

### *Prevalence of species of strains of the genus Acinetobacter isolated*

**Figure 1:**
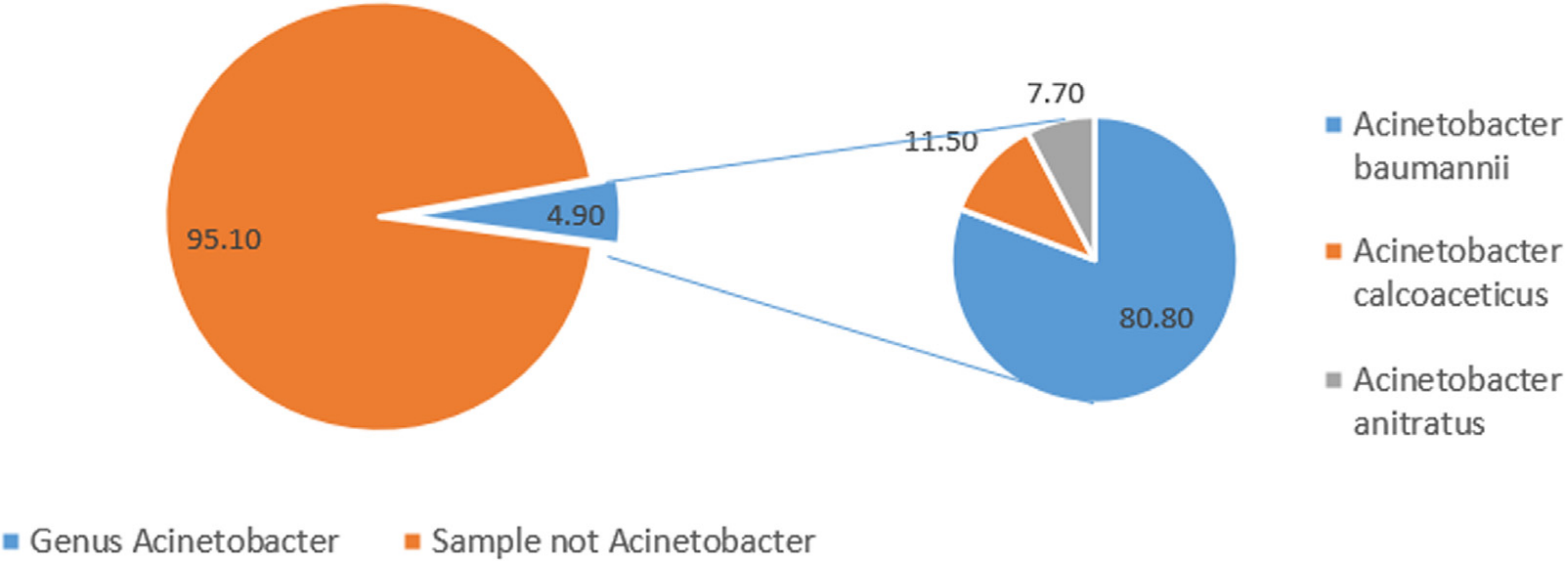
Prevalence of the *Acinetobacter* genus in the study population. This chart illustrates the proportion of samples containing *Acinetobacter* (4.9%) among the 531 clinical samples collected. Among these isolates, *Acinetobacter baumannii* (light blue) accounted for 80.8%, followed by *Acinetobacter calcoaceticus* (orange) at 11.5% and *Acinetobacter anitratus* (gray) at 7.7%. The remaining samples (dark orange, 95.1%) did not contain any *Acinetobacter* species.

Among the 531 biological samples collected, 26 (4.9%) species belonging to the *Acinetobacter* genus were isolated. However, *Acinetobacter* represented a minority of all bacterial isolates processed during the study period, indicating a relatively limited overall clinical burden compared with more common pathogens (data not shown). Among these, 80.8% were *A. baumannii*, followed by *A. calcoaceticus* (11.5%) and *A. anitratus* (7.7%) (Figure 1).

### *Prevalence of carbapenemase-producing Acinetobacter species*

**Figure 2:**
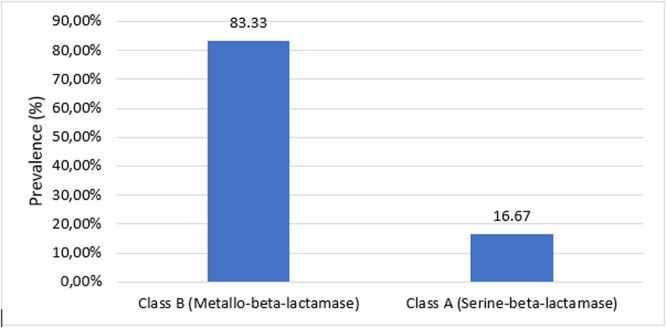
*Acinetobacter baumanii*-producing carbapenemase. This bar chart shows the prevalence of carbapenemase types identified in *Acinetobacter baumannii* isolates. Class B enzymes (metallo-beta-lactamases) were the most common, detected in 83.33% of cases, whereas class A enzymes (serine-beta-lactamases) were found in 16.67%. Metallo-beta-lactamases hydrolyze beta-lactams through zinc-dependent mechanisms, whereas class A enzymes use serine at their active site.

The production frequency of carbapenemase and the classification of carbapenemases are shown in Figure 2. In this study, the overall prevalence of *A. baumannii*-producing carbapenemase was 85.71% (18/21), including 83.33% (15/18) of class B (MBL) and 16.67% (3/18) of class A.

### *Susceptibility of carbapenemase-producing Acinetobacter species isolates to beta-lactams and other families of antibiotics*

The sensitivity profile of *A. baumannii* to beta-lactams is presented in Table 2. The resistance profile of *A. baumannii* showed a very high resistance rate to all beta-lactams, including penicillins (*piperacillin*: 94.44% and *ticarcillin*: 94.44%), cephalosporins (*cefotaxime*: 77.78%, *ceftazidime*: 88.89%, and *cefepime*: 77.78%), and carbapenems (*imipenem*: 66.67%). In addition, a high resistance rate was observed for beta-lactams combined with beta-lactamase inhibitors (*piperacillin/tazobactam*: 83.33% and *ticarcillin/clavulanic acid*: 83.33%). The resistance profile of *A. baumannii* to aminoglycosides, fluoroquinolones, tetracyclines, polymyxins, and cotrimoxazole was tested to determine its resistance pattern (Table 2). A high resistance rate was observed for fluoroquinolones (*levofloxacin*: 94.44%), tetracycline (66.67%), and aminoglycosides (*tobramycin*: 77.78%, *gentamicin*: 61.11%, and *amikacin*: 44.44%). However, resistance to colistin was low (11.11%).

**Table 2:** Susceptibility of carbapenemase-producing *Acinetobacter* species isolates to beta-lactams.

Susceptibility of carbapenemase-producing *Acinetobacter* species isolates to beta-lactams								
	IMP N (%)	CEF N (%)	CAZ N (%)	COT N (%)	TIC/CLA N (%)	TIC N (%)	PIP/TAZ N (%)	PIP N (%)
Sensitive	6 (33.33)	4 (22.22)	2 (11.1)	4 (22.22)	3 (16.67)	1 (5.56)	3 (16.67)	1 (5.56)
Resistant	12 (66.67)	14 (77.78)	16 (88.9)	14 (77.7)	15 (83.33)	17 (94.4)	15 (83.33)	17 (94.44)

Susceptibility of carbapenemase-producing *Acinetobacter* species isolates to other families of antibiotics								
	COL N (%)	TRI/SUF N (%)	TET N (%)	LEV N (%)	TOB N (%)	GEN N (%)	AK N (%)	-

Sensitive	16 (88.89)	5 (27.78)	6 (33.33)	1 (5.56)	4 (22.22)	7 (38.89)	10 (55.56)	-
Resistant	2 (11.11)	13 (72.22)	12 (66.67)	17 (94.44)	14 (77.78)	11 (61.11)	8 (44.44)	-

AK: amikacin; CAZ: ceftazidime; CEF: cefepime; CLO: colistin; COT: cefotaxime; GEN: Gentamicin; IMP: imipenem; LEV: levofloxacin; PIP/TAZ: piperacillin/tazobactam, PIP: piperacillin; TAC/CLA: ticarcillin/clavulanic acid; TET: tetracyclin; TIC: ticarcillin; TRI/SUF: trimethropim/sulfamethoxazol; TOB: Tobramycin.

### *Risk factors associated with carbapenemase infections*

Multivariate analysis of risk factors for carriage of carbapenemase-producing *A. baumannii* in infected participants is presented in Table 3. This revealed that the age group 38-47 years (OR = 0.075; 95% CI 0.006-0.936, *P*-value = 0.044), hospitalization of patients (OR = 10. 50; 95% CI 2.25-58.18, *P*-value = 0.001), antibiotic therapy (OR = 6.85; 95% CI 1.17-29.50, *P*-value = 0.035), and use of antibiotics in the last 3 months (OR = 7.67; 95% CI 4.17-19.50, *P*-value = 0.004) were negatively associated with carriage of carbapenemase-producing *A. baumannii* in infected patients. These results concern infections caused by *A. baumannii*, which was predominantly isolated.

**Table 3:** Multivariate analysis of risk factors related to carbapenemase producing *Acinetobacter* in hospitalized patients.

	Adjusted odds ratio (95% confidence interval)	*P*-value
**Sex**		
Female	1	Ref
Male	0.231 (0.040-1.347)	0.103
**Age group (years)**		
18-27	1	Ref
28-37	0.250 (0.17-3.660)	0.311
38-47	0.075 (0.006-0.936)	**0.044**
>48	0.42 (0.07-2.54)	0.267
**Marital status**		
Single	1	Ref
Married	0.600 (0.109-3.296)	0.557
**Level of education**		
Primary	1	Ref
Secondary	1.22 (0.158-9.467)	0.848
University	14.50 (3.13-28.18)	0.967
**Type of income**		
Seasonal	1	Ref
Permanent	8.750 (0.884-86.603)	0.064
**Hospitalized patient**		
No	1	Ref
Yes	10.50 (2.25-58.18)	**0.001**
**Currently on antibiotics**		
No	1	Ref
Yes	6.85 (1.17-29.50)	**0.035**
**Have you used antibiotics in the last 3 months?**		
No	1	Ref
Yes	7.67 (4.17-19.50)	**0.004**
**Have you been hospitalized in the last 6 months?**		
No	1	Ref
Yes	1.14 (0.02-3.41)	0.201

## Discussion

In an effort to address the emergence and spread of multidrug-resistant bacteria and to develop targeted strategies for controlling infections caused by carbapenemase-producing *Acinetobacter* species in Yaoundé, we conducted this study. Our objective was to determine the prevalence of carbapenemase-producing *Acinetobacter* species and the associated risk factors in selected health care facilities in Yaoundé, Cameroon.

Among the 531 samples analyzed, 26 isolates belonging to the *Acinetobacter* genus were identified, representing a crude prevalence of 4.9%. This proportion is reported here relative to the total number of samples. However, for a more relevant estimation of the relative prevalence of *Acinetobacter*, it would have been more rigorous to relate these isolates to the total number of all bacterial strains identified in the same samples. Due to the lack of systematic recording of all isolated pathogens, this comparison could not be performed. This represents a methodologic limitation that should be addressed in future studies. Among the *Acinetobacter* genus isolated, 80.8% were identified as *A. baumannii*, followed by *A. calcoaceticus* (11.5%) and *A. anitratus* (7.7%). Although the study included different species, the results were mainly dominated by *A. baumannii*. It would be wise to adjust the conclusions accordingly or to further compare the resistance profiles of secondary species.

This distribution aligns with the findings of Djuikoué et al. [16] in Cameroon, who reported a predominance of *A. baumannii* in local nosocomial infections. Globally, *A. baumannii* is the primary species associated with nosocomial infections [2]. However, the relatively low prevalence of *Acinetobacter* species in our study may be influenced by several factors, including the types of samples analyzed, the clinical context of patients, infection control practices, competition with more common bacteria, and the experimental conditions used for strain isolation.

Among the 21 *A. baumannii* isolates, 85.71% were identified as carbapenemase producers, with 16.67% belonging to class A carbapenemases and 83.33% to class B. This prevalence is higher than the rates reported by Djiukwo et al. [16] (40.12%) and Mizan et al. [20] (56.97%) [16,20]. The observed difference could be attributed to high local antibiotic pressure, variations in methodology, the circulation of resistant clones, and differences in geographical or institutional approaches to managing nosocomial infections. MBL was the most frequently found enzyme among the carbapenemase-producing *A. baumannii* isolates, a finding consistent with global studies [21]. Carbapenemases, classified into three beta-lactamase classes, can hydrolyze all beta-lactam antibiotics and carry additional antibiotic resistance genes [22]. The spread of MBL gene-carrying strains is a concern, and recent studies in Cameroon have raised alarms about the production of beta-lactamases in certain bacterial species [23].

In this study, most participants were men (54.4%), with the 18-27 age group being the largest (48.8%), consistent with other African studies highlighting young adults' vulnerability to nosocomial infections [6]. Despite 68.5% having university-level education, access to health care and infection prevention awareness remained limited [16]. In addition, 71% were single and 74.4% had seasonal incomes, indicating economic instability that may encourage self-medication, a known driver of antibiotic resistance [8].

The study showed that 25.8% of patients were hospitalized, mainly in internal medicine (75.2%), aligning with findings in sub-Saharan Africa where *A. baumannii* affects chronically ill, long-term patients [14]. Antibiotic use was high (37.7%), with ceftriaxone being most prescribed (79.5%). This raises concerns about resistance, especially in *Acinetobacter spp.*, known for developing multidrug resistance [5].

The study found that 6.2% of patients had comorbidities, with diabetes being the most common (63.6%), supporting research that links diabetes to increased susceptibility to *A. baumannii* infections [2]. In addition, 47.6% had used antibiotics recently, mainly cefepime (30.4%), ceftriaxone (22.9%), and ciprofloxacin (16.9%), a known risk factor for resistant strains [3]. Moreover, 19.8% had been hospitalized within 6 months, all exposed to invasive devices like catheters and ventilators, which facilitate the spread of resistant nosocomial infections such as those caused by *A. baumannii* [13].

These findings highlight the need for increased surveillance of nosocomial infections and rational use of antibiotics, by limiting over-prescription of cephalosporins and fluoroquinolones and improving hospital hygiene protocols [11]. Our results also highlight the importance of revising empirical treatment protocols in high-risk departments and strengthening antibiotic stewardship programs.

In this study, 35.8% of strains were isolated from midstream urine, 13.4% from urethral, and 11.9% from cervico-vaginal samples. These findings contrast with Djuikwo et al*.* [16], who identified suppurations, urine, and blood as primary sources. Differences may stem from variations in patient profiles. Notably, only 1.3% of catheter-associated urinary tract infections were linked to catheter tips, despite their known role in promoting bacterial colonization and catheter-associated urinary tract infections in health care settings [24].

Based on antimicrobial susceptibility testing, resistance rates to the tested antibiotics tested ranged from 44.44-94.44%, except for colistin, which exhibited a resistance rate of 11.11%. The colistin resistance rate (11.1%) observed in this study was assessed using the E-test method for determining the MIC, in accordance with the manufacturer's recommendations. No molecular confirmation by polymerase chain reaction (search for mcr genes) was performed, which is a limitation in terms of confirming with certainty the genetic mechanism underlying this resistance. Carbapenem resistance in *A. baumannii* is a major concern, as these antibiotics are the preferred treatment option for infections caused by this bacterium [25].

The results showed that *A. baumannii* exhibited a very high rate of resistance to beta-lactams, with resistance rates of 88.89% for ceftazidime and 77.78% for both cefepime and cefotaxime. In addition, resistance to carbapenems was noted, with imipenem resistance at 66.67%. A study by Djiukwo et al. [16] corroborated these findings, reporting resistance rates of 82.9% for ceftazidime, 74.1% for cefepime, and 61.7% for imipenem [16]. Similar high resistance rates were documented in South Africa, with 73% resistance to cefepime and 80% to ceftazidime [26]. Furthermore, Anane et al. [27] reported exceptionally high resistance rates to beta-lactams, particularly imipenem (81%) and meropenem (83%) [27].

Comparing our findings with those from previously mentioned studies reveals that the resistance rate of resistance of *A. baumannii* to antibiotics has not decreased significantly, but third- and fourth-generation cephalosporins and carbapenems are becoming increasingly ineffective in treating infections in Cameroon, which poses a problem [12].

Antibiotic susceptibility tests revealed high resistance in *A. baumannii*, particularly to fluoroquinolones (94.44% for levofloxacin), tetracyclines (66.67%), and several aminoglycosides—tobramycin (77.78%), gentamicin (61.11%), and amikacin (44.44%). Resistance to trimethoprim/sulfamethoxazole was 72.22%. Amikacin showed lower resistance than other aminoglycosides. These rates exceed those reported in previous Cameroonian studies [16,17], suggesting an increase in resistance likely due to the widespread use of fluoroquinolones. This highlights the evolving threat of drug-resistant *A. baumannii*.

Resistance to fluoroquinolones in Gram-negative bacteria, particularly *A. baumannii*, is a growing concern due to excessive and inappropriate use on the pharmaceutical market [28].

Managing the burden of *A. baumannii* is challenging due to its ability to easily acquire antimicrobial resistance genes, facilitated by its flexible genome [22]. Compared with Anane et al. [27], who reported a 5% resistance rate to colistin [27], this study found an increase in colistin resistance rates. However, *A. baumannii* remains sensitive to colistin, suggesting that it is still a viable option for treating infections caused by this bacterium in Cameroonian health care facilities. Colistin monotherapy faces significant management challenges due to nephrotoxicity, neurotoxicity, and potential resistance, necessitating continued monitoring and development of new therapeutic alternatives [22].

The analysis of risk factors revealed that the age group of 38-47 years (OR = 0.075; *P*-value = 0.044), recent hospitalization (OR = 10.50; *P*-value = 0.001), current antibiotic therapy (OR = 6.85; *P*-value = 0.03), and antibiotic use within the past 3 months (OR = 7.67; *P*-value = 0.004) were significantly associated with the carriage of resistant *A. baumannii.* These findings align with the study by Murray et al. [2], who identified prolonged hospital exposure and frequent antibiotic therapy as major risk factors for A. baumannii infection [2]. Furthermore, research conducted by Eze et al. [29] in South Africa indicated that hospital effluents represent a potential risk for the formation of multi-resistant biofilms by *A. baumannii* strains [29]. *A. baumannii*’s significant propagation and prevalence in hospitals are facilitated by its survival in cold and humid environments, resistance to disinfectants and antibiotics, and its ability to form biofilms [30].

This study has limitations, including the lack of molecular characterization of carbapenemases and a small sample size for non-*baumannii Acinetobacter* species, restricting species-specific analysis. However, it is the first comprehensive investigation in Yaoundé on carbapenemase-producing *Acinetobacter*, combining clinical, microbiologic, and epidemiologic data. It provides essential baseline data on resistance and colistin susceptibility, supported by validated methods and strict quality control. The findings enhance local knowledge on resistance and aid infection control and antimicrobial stewardship in Cameroonian hospitals.

## Conclusion

This study highlights the concerning prevalence of carbapenemase-producing *Acinetobacter* species in certain health care facilities in Yaoundé, with a high presence of *A. baumannii* and high resistance to antibiotics, particularly carbapenems. Our results emphasize the urgent need for enhanced epidemiologic surveillance and rigorous antibiotic management to curb the spread of these highly resistant bacteria. Identifying risk factors such as recent hospitalization and antibiotic use could contribute to developing tailored prevention and control strategies. Collaborative efforts among health care professionals, health authorities, and researchers are essential to strengthen biosafety measures in hospitals and promote the rational use of antibiotics. Given the relatively low proportion of *Acinetobacter* among total isolates and the potential inclusion of colonizing strains, our findings should be considered exploratory and context-specific. Moreover, further studies, including in-depth molecular characterization of the isolated strains, are necessary to gain a better understanding of resistance mechanisms and to propose alternative therapeutic solutions.

## Declarations of competing interest

The authors have no competing interests to declare.
